# Tin and Its Combinations with Gold and Amalgam

**Published:** 1895-04

**Authors:** S. B. Palmer

**Affiliations:** Syracuse, N. Y.


ARTICLE II.

### TIN AND ITS COMBINATIONS WITH GOLD AND AMALGAM.

BY S. B. PALMER, M. D. S., SYRACUSE, N. Y.

Tin has been used for a long time, and its merits fully recognized. It is soft, easily adapted to the walls of the cavity, is the most compatible with dentine of any of the metals used, is a poor conductor of thermal changes, it oxidizes but slightly, while the oxidation between the filling and walls of the cavity is insoluble, filling the dental tubuli and decalcified dentine and thus arresting further decay. This constitutes the antiseptic properties claimed for tin. But it fails to meet all the conditions for permanency. It is non-cohesive, and when packed in a cavity in layers it will flake.

Some years ago, Dr. Frank French, of Rochester, wrote me, asking for the chemical relations or combinations of tin and gold. I could not answer, nor have I found a solution, till by observation and experiments I have settled on the following as scientific and correct. These three effects are visible:

First. Tin and gold unite in fillings, and there is no separation at the union.

Second. Tin and gold work well together where tin comprises most of the filling, and gold is added as a protection from wear, or for appearance.

Third. When tin is used as a guard filling, under gold, or as a lining, and the tin exceeds in thickness three leaves of foil, a portion of the tin next the dentine will be odidized and remain a soft black paste, no decay being found so long as the oxid remains, but as it is too soft for the brush, tooth-pick, etc., it is unreliable.

Thus we have three distinct effects and corresponding conditions. Let us look for the causes. The one great hindrance to progress in dental science has been failure to find the source of each particular effect.

First. The union of tin and gold in the mouth. The two metals must be in contact. The process by which the metals unite is electrolysis. It is a law of electrolysis that no single element can be decomposed; also that fluidity is essential for molecular movements. When, therefore, gold and tin are united or compacted in a filling, the two metals within remain distinct and unchanged. But on the surfaces exposed to moisture, even to that in porous dentine, the tin is oxidized by reason of the current set up by the gold. Thus the tin is dissolved, and by the current, as in the electro-plating bath, the atoms of tin are attached to and fastened on the gold, similar to plating gold with tin. The tin plating thus formed will not exceed the thickness of two leaves of foil.

To make this plain: If mercury is placed on gold or silver it will amalgamate till the mercury is satisfied, and it will go no deeper unless more mercury is added. Mercury is a metal in a state of fusion, while the tin is held in a liquid state. The tin does not penetrate the gold to any great depth, but the gold influences the tin by mutual induction, as zinc affects iron to render it negative. Thus an amount of tin is preserved to the thickness of two folds of tin foil. There can then be no separation of tin and gold, because the tin which is united with the gold is negative to the other portions of the tin. When tin constitutes a portion of the filling, the two metals work well, as the tin is but slightly oxidized and not dissolved by galvanic action. But when only three or four thicknesses of tin are used, the one or two leaves which will be in excess of the preserving influences of the gold, with no body of tin back of it, are in danger of its losing their integrity as a metal, and becoming dissolved as before mentioned. This difficulty may be obviated by mixing the metals in the foils by alternate layers, forming the leaves into tape blocks, or rolled into cylinders. Thus guard fillings or whole fillings may be made, which have many advantages over amalgam. The alloy thus formed resembles amalgam in color and hardness, does not shrink, and preserves dentine as well as tin alone. Any amount of gold may be used without injury, and the excess which is not taken up in forming the alloy will show the gold bright and clear.

On the other hand, an excess of tin will be marked by lines or pits in the filling showing where tin has been dissolved. It has been mentioned that this chemical action occurs only on the surfaces exposed to moisture. I will add that in building up large fillings, it is important to have the fillings wet when inserted; then the whole plug is as solid as amalgam. The moisture sets up electro-chemical action, with the results already stated.*

One more statement in relation to tin and gold, and we will take up the next combination. About thirty-seven years ago I filled the upper incisors for a lady patient who could not afford the money to pay for gold, while I could not afford the reputation to use amalgam. The cavities were proximate, and the enamel on lingual surfaces badly broken away, with the labial surfaces transparent, yet well preserved. The cavities were lined with non-cohesive gold and filled with tin. I saw the fillings several years after. The gold was visible through the enamel and the fillings were perfect, no appearance of recurring decay. This case with others was reported at a society meeting, and probably published. I quote the following from the remarks of Dr. C. S. Stockton, Newark, N J., at the World's Columbian Dental Congress:

"I recall two fillings that I saw only a short time since, which were put in twenty-three years ago, I think. I filled those teeth by a plan recommended by Dr. Palmer,

---

*Since writing the above, I placed a layer of tin foil on a plata of 22k. gold and inclosed it in plaster, with a case, while vulcanizing. The two metals were united, forming an alloy of gold and tin.

using mats of Abby's soft foil packed up against the labial surfaces of the enamel, filling the remainder of the cavity with tin foil. They are in as good condition to-day as they were twenty-three years ago."

Now let us study the relations of tin and amalgam. This we cannot do without a knowledge of some of the properties of amalgam not given in the books. A few words will excuse me from lengthy remarks on copper amalgam, which I have used only experimentally. The teachings of nature's simplest text-book, the galvanic battery, would enable any one familiar with electrical science to know that an amalgam composed of two single elements could not be relied on. Copper and mercury are brought together and form a hard mass, still each metal retains its individuality. Heat the mass and the mercury will be partially expelled, the compound becoming plastic, which is not the case with an alloy of two or more metals. The relations of copper and mercury are the same as copper and zinc in a battery, only that there is no great electrical difference or potential between copper and mercury. Like saliva, the positive and negative relations are liable to change. At times the copper becomes the positive element; that is, the fluids may oxidize copper more readily than mercury, when the filling will appear white from excess of mercury on the surface, and the mercury is soon worn away. When the affinity of the matter in the mouth is stronger for mercury, the filling appears brown and spongy, and is too soft to stand abrasion. When the action is equal, or so nearly so that the oxidation on the surface unites to form a black, hard compound, then one metal protects the other, and copper amalgam is pronounced a success.

The setting of ordinary amalgam can be well understood by the process already described in the combination of tin and gold. In amalgamating the fillings of the alloy; mercury is absorbed, or enters into the alloy, according to the fineness of the cutting. The excess of mercury is forced out, and the filling inserted. The amalgamation of the mass is by no means completed, but continues till the mercury has been taken up and firmly held in combination. Then it is that the mass has become hard. This process requires more or less time, for two prominent reasons, the age of the alloy, and the thickness of the fillings or shavings. Fine fillings, when first cut, will set nearly as quick as oxiphosphates.

Let us now consider the condition of an amalgam filling which has gained its maximum degree of hardness. It is not an alloy, as if all the metals were fused by heat. There are two distinct conditions in an amalgam plug. There are the coarser particles, the center of which contains less mercury than the other portions, and there is the bulk of the mass, which is thoroughly amalgamated. In all high grade amalgams, mercury is the positive element, the one to be most acted on. We said that in the combination of tin and gold, where there is an excess of gold, it remains distinct, so that the unamalgamated particles are negative to the portions containing more mercury. But there is also local electric action going on on the surfaces of all amalgam fillings that do not protect themselves by sulphates or oxides of the metals.

Amalgams which will stand the tests for shrinkage perfectly, if filled on soft dentine for a few years, on removal will appear rough, blackened, and show none of the fine lines of adaptation to the dentine that they would have done on removal at first. This is according to a well-known law, which can only be set aside by changing conditions. The cause of shrinkage and failure of amalgams, after many years, is from galvanic action between the unequally amalgamated portions of the plug and the other portions containing more mercury, much the same as between copper and mercury. The rough surface of an old amalgam filling is caused by the dissolving of the mercury by local currents, generated by the potential difference between the positive and negative relations between the metals. This principle is the great cause of failure, and we have, to a considerable extent, corrected the difficulty in the use of copper in the alloy, depending on oxidation to change the positive relations of the dentine to negative; but when the local action is so vigorous as to produce acid, the lime constituents of the dentine are attacked, and the same results follow as in the use of gold.

Study of a single galvanic battery affords a lesson, and I think a remedy for this difficulty, by which we may use amalgam without copper. The lesson is this: Commercial zinc contains other metals, among which iron is prominent. With pure zinc in given liquids, used in some forms of batteries, there would be no consumption of zinc with an open circuit; but with commercial zinc with an open circuit there is local action, and the zinc is dissolved without contributing to the general current. That is analagous to an amalgam filling. There is local action and consumption, which are often taken for shrinkage, the cavity being also enlarged.

The remedy adopted to prevent local action on zinc is to amalgamate the plate, which neutralizes all local differences, only one metal—mercury—coming to the surface. Reasoning from this fact and knowing another quite as practical, that tin fillings do not shrink, and that no secondary decay appears, then we can use amalgam free from copper by lining cavities with tin foil. I know that local action is done away with, that shrinkage cannot occur, that dentine will not be dissolved, and I believe by this that we get the combined benefits of tin for tooth preservation, and of amalgam to withstand attrition.

Another aspect of amalgam fillings is when two or more pieces have been joined at different times. After years the line of junction will be found distinct and depressed, and occasionally separation is complete. The cause is this: That portion which acts as solder to unite the pieces contains more mercury than the filling on either side; consequently, it becomes a thin, positive element, between two elements negative to it, with results as stated. The remedy consists in amalgamating the surface of the old amalgam, and covering it with a layer of gold foil, filled on the gold, which at once disappears. Joints thus treated become negative to the fillings proper; consequently, the union is preserved.

In closing, we mention combinations of gold and amalgam. The principles are the same as those already mentioned; therefore, little more need be said to make the application. When gold and amalgam are to be used in the same tooth, by all means unite the two, or there will be a current passing from one to the other through any conductor that reaches, from one to the other. Any sensitive person can realize this by covering with the tip of the tongue the two fillings. When the connection is made by uniting the fillings, no current will leave the metal to effect the taste or dentine, and the union of gold and amalgam is ever perfect, because the mercury takes up gold enough to make the joint negative to the amalgam, so that if the fillings were placed in dilute nitric acid after the amalgam should be dissolved, that layer which composed the joint would be found intact with the gold. Amalgam when connected with gold becomes positive, and, consequently, shows oxidation to blackness. This, however, is a benefit, inasmuch as it greatly overcomes local action, which has already been mentioned.

Amalgam is good to use as a guard filling under gold, and particularly in proximate cavities in bicuspids, where the cavity extends up too far to allow the application of the rubber-dam. Amalgam may be used to fill the root portion, and, when set, the dam can be applied, and gold used to complete the filling. On account of the local currents which are present with amalgam, I much prefer tin prepared as before mentioned. There is much more disturbance between two proximate amalgam fillings than between two of gold; therefore it is better to fill with tin, or tin and gold, at the cervical border, and finish with amalgam, because tin is a poor conductor as compared with gold or amalgam. According to Cassidy, the conductivity of the principal metals are rated thus:

| | |
|---|---|
| Silver, | 1,000 |
| Copper, | 736 |
| Gold, | 532 |
| Tin, | 145 |
| Iron, | 119 |
| Lead, | 85 |
| Platinum, | 84 |
| Dentine (normal moist), | 15 |

Amalgam is not given, but from the silver which enters into its composition it would probably be above tin.—*Items of Interest.*

